# Comparative Effectiveness of Devices for Interventional Patent Foramen Ovale Closure: Insights from a 23-Year Monocentric Analysis

**DOI:** 10.3390/jcm13216354

**Published:** 2024-10-23

**Authors:** Elham Kayvanpour, Elena Matzeit, Ziya Kaya, Sven Pleger, Anke Bahrmann, Christine Reichardt, Peter Arthur Ringleb, Norbert Frey, Benjamin Meder, Farbod Sedaghat-Hamedani

**Affiliations:** 1 Department of Internal Medicine III, Heidelberg University, 69120 Heidelberg, Germany; elham.kayvanpour@med.uni-heidelberg.de (E.K.); benjamin.meder@med.uni-heidelberg.de (B.M.); 2 DZHK (German Center for Cardiovascular Research), Partner Site Heidelberg and Mannheim, 69120 Heidelberg, Germany; 3 Department of Neurology, Heidelberg University, 69120 Heidelberg, Germany; christine.reichardt@med.uni-heidelberg.de (C.R.);; 4 Department of Genetics, Stanford Genome Technology Center, Stanford University School of Medicine, Stanford, CA 94305-5101, USA

**Keywords:** PFO closure, cryptogenic stroke, transient ischemic attack, residual shunt, new-onset atrial fibrillation

## Abstract

**Background/Objectives:** Patent foramen ovale (PFO) is a congenital heart defect affecting up to 25% of the population, associated with an increased risk of cryptogenic stroke. Percutaneous PFO closure is a minimally invasive procedure aimed at reducing stroke risk by eliminating the right-to-left shunt. **Methods:** This monocentric, retrospective study analyzed 716 patients who underwent PFO closure between January 2000 and February 2023. Data collected included demographics, indications for closure, procedural details, and outcomes. Key endpoints were complications at the puncture site, pericardial effusion, recurrent stroke or transient ischemic attack (TIA), thrombi on the device, new-onset atrial fibrillation, and death. **Results:** The cohort had a mean age of 50.6 ± 12.6 years, with 60.8% female. Four devices were used: Occlutech PFO occluder (*n* = 106), Amplatzer PFO occluder (*n* = 227), Gore septal occluder (*n* = 296), and Cardia PFO-Star (*n* = 87). The initial procedural success rate was 98.9%, with no significant differences between devices. Complication rates were low across all devices. Residual shunt incidence decreased from 17.9% in 1 month to 3.4% in 12 months. Device-specific complications included late-onset pericardial effusion (*p* = 0.01), erosions (Cardia PFO-Star), and device thrombus formation (Gore septal occluder). **Conclusions:** PFO closure is a safe and effective method for preventing recurrent strokes, with high success rates and varying specific complication profiles, depending on the device. Further long-term studies are needed to evaluate newer devices and optimize patient outcomes.

## 1. Introduction

Patent foramen ovale (PFO) is a common congenital heart defect, occurring in up to 25% of the general population [1]. It is a remnant of fetal circulation, where the foramen ovale, a flap-like opening between the right and left atria, fails to close after birth. In the majority of individuals, the foramen ovale closes spontaneously within the first year of life. However, in a significant proportion of the population, the foramen ovale remains patent, creating a potential right-to-left shunt.

The prevalence of PFO is nearly twice as high in patients with cryptogenic stroke compared to the general population [1]. Although the risk of recurrent stroke is generally low even in patients who do not undergo PFO closure (referred to as nonclosure-arms in clinical studies), research indicates that this risk increases with age [2]. This underscores the need for careful consideration of PFO closure, particularly in younger patients who may benefit more from the procedure. Additionally, atrial fibrillation, left atrial thrombus, and aortic atheroma are common causes of cardioembolism that must be excluded before attributing a stroke to PFO. Non-invasive imaging techniques, such as transthoracic echocardiography (TTE), transesophageal echocardiography (TEE), and cardiac magnetic resonance (CMR), are essential for detecting potential sources of embolism. Recent advancements in imaging have enhanced cardiovascular risk assessment, allowing for more accurate identification of high-risk conditions [3]. These technologies are crucial in excluding alternative causes of embolism and optimizing preventive strategies in patients undergoing PFO closure.

Percutaneous PFO closure is a minimally invasive procedure that deploys a device to occlude the PFO, effectively eliminating the potential right-to-left shunt and reducing the risk of paradoxical embolism leading to cryptogenic stroke. Various occluder devices, including umbrella, disc, and bioabsorbable designs have been developed, each with its own unique design and characteristics [4]. Their safety and feasibility have been demonstrated in several systematic reviews and meta-analyses of randomized controlled trials [5,6,7].

Given the potential benefits of PFO closure and the variety of available devices, there remains a need for further observational studies to evaluate long-term outcomes and procedural efficacy in real-world clinical settings. Additionally, newer devices and models that have not yet been studied in randomized controlled trials are now being used, and their performance and safety need to be assessed. Therefore, we conducted a mono-centric observational study with a retrospective cohort and a 12-month follow-up to gain deeper insights into the clinical outcomes associated with percutaneous PFO closure and to assess the performance of different occluder devices used in routine practice. This study aims to contribute valuable data to inform clinical decision-making, evaluate the efficacy of newer occluder devices, and optimize patient care.

## 2. Materials and Methods

### 2.1. Study Design and Population

This was a retrospective, observational study conducted at a single tertiary care center. The study included 716 patients who underwent percutaneous PFO closure between January 2000 and February 2023. The study was approved by the local ethics committee and was conducted in accordance with the Declaration of Helsinki. Informed consent was waived due to the retrospective nature of the study. Patients were identified from the institutional PFO registry and were included in the study if they were 18 or older, at the time of implantation.

### 2.2. Data Collection

Data were collected from the electronic health records of the included patients. The following data were collected: patient demographics, indication for PFO closure, shunt before and after the intervention, laboratory values (including renal parameters, troponin, and NT-proBNP), ECG parameters, transthoracic and transesophageal echocardiography parameters (including shunt evaluation and device-related thrombus formation), medication at discharge, peri- and post-interventional complications, and follow-up data, which were collected at 1 month, 6 months and 12 months post-intervention.

### 2.3. PFO Closure Procedure and Device Selection

Percutaneous PFO closure was performed by 10 experienced interventional cardiologists using standard techniques. The procedures were performed under conscious sedation. The choice of PFO closure device was at the discretion of the treating physician, based on patient anatomy, device characteristics, and the physician’s expertise. The procedures were guided using both fluoroscopy and transesophageal echocardiography (TEE) to ensure accurate positioning of the devices and to monitor for potential complications during the procedure. The devices used in this study included the Occlutech PFO occluder (Occlutech GmbH, Jena, Germany), Amplatzer PFO occluder (Abbott Laboratories, St. Paul, MN, USA), Gore septal occluder (W. L. Gore & Associates, Inc., Flagstaff, AZ, USA), and the Cardia PFO-Star (Cardia, Inc., Eagan, MN, USA).

### 2.4. Outcome Measures

Outcome measures were the incidence of recurrent stroke or transient ischemic attack (TIA) after PFO closure during the follow-up period as well as the incidence of peri- and post-interventional complications, changes in echocardiographic parameters (such as residual shunt, device position, and device-related thrombus formation), and new-onset atrial fibrillation/flutter as assessed by electronic health records.

### 2.5. Statistical Analysis

Descriptive statistics were used to summarize patient demographics, device characteristics, and outcome measures. Categorical variables were presented as frequencies and percentages, while continuous variables were presented as means and standard deviations or medians and interquartile ranges, as appropriate. Comparisons between different PFO closure devices were performed using appropriate statistical tests, such as ANOVA or Kruskal–Wallis test for continuous variables, and chi-square or Fisher’s exact test for categorical variables. A *p*-value of <0.05 was considered statistically significant.

## 3. Results

### 3.1. Patient Characteristics and Demographics

A total of 716 patients (mean age 50.6 ± 12.6 years, 60.8% male) underwent percutaneous PFO closure during the study period, using four different occluder devices: Occlutech PFO occluder (*n* = 106), Amplatzer PFO occluder (*n* = 227), Gore septal occluder (*n* = 296), and Cardia PFO-Star (*n* = 87). There were no significant differences between the groups regarding age, BMI, gender, or cardiovascular risk factors (Table 1). The most common indications for PFO closure were cryptogenic stroke (66.8%), TIA (18%), and peripheral embolism (6%) (Table 2). Transesophageal echocardiography (TEE) revealed a hypermobile septum in 41.5% of patients and an atrial septal aneurysm (ASA) in 29.6% of patients. The mean ASA size was 13.4 ± 3.9 mm.

### 3.2. Procedural Data and Periprocedural Complications

The overall initial procedural success rate was 98.9%, indicating that the vast majority of PFO closure procedures were completed without major technical difficulties. The initial success rates for implantation did not significantly differ between device types, being 100% for Occlutech PFO occluder, 97.8% for Amplatzer PFO occluder, 99.3% for Gore septal occluder, and 98.9% for Cardia PFO-Star. From eight initially unsuccessful device implantations, two could be implanted in a second session. The unsuccessful implantations were mainly due to patient restlessness, inability to advance the occluder into the left atrium, inadequate septal rims or unfavorable PFO morphology, and cases where transesophageal echocardiography (TEE) was not possible due to patient intolerance. These factors posed challenges to the safe completion of the procedure. No significant differences were found between the devices regarding the average total procedure duration (36.2 ± 17.8 min). Although the fluoroscopy duration differed significantly between devices, it remained very short for all. The longest duration was observed with the Occlutech PFO occluder device (5.9 ± 5.3 min), while the shortest was with the Cardia PFO-Star (3.6 ± 3.0 min) (Table 3). Following PFO closure, dual antiplatelet therapy (aspirin and clopidogrel) was prescribed for a period of one month. Lifelong aspirin therapy was prescribed if patients had additional risk factors or in cases of a residual shunt.

Periprocedural complications were vascular access site complications (0.6%), new-onset atrial fibrillation (0.6%), and hemodynamically significant pericardial effusion (0.3%). TIA and device embolization happened in 0.1% of cases each. Air embolism was suspected in one patient due to an inadequate awakening response after PFO closure. The suspected diagnosis was confirmed by a cranial CT, and the patient was intubated and received hyperbaric oxygen therapy in a pressure chamber. The periprocedural complication rates were similar across the different PFO closure devices, suggesting that the choice of device did not significantly impact the procedural safety profile (Table 4).

### 3.3. Residual Shunt (RLS)

**Figure 1:**
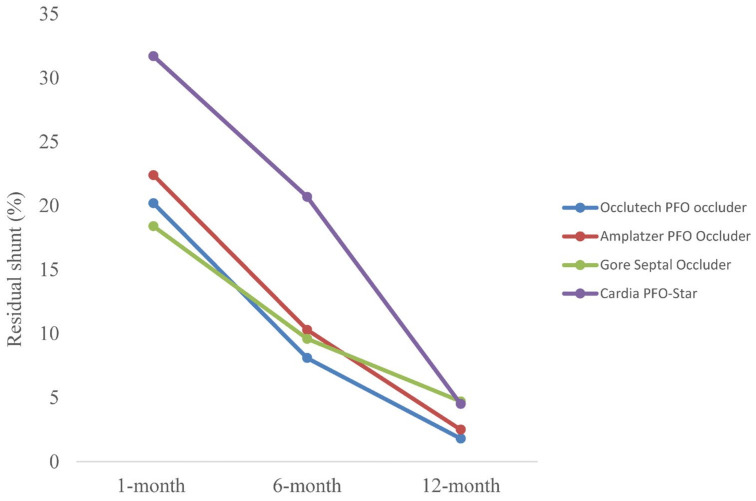
Residual right-to-left shunt (RLS) rates following PFO occluder implantation for different devices at 1-month, 6-month, and 12-month follow-ups. The Occlutech PFO occluder (blue), Amplatzer PFO Occluder (red), Gore Septal Occluder (green), and Cardia PFO-Star (purple) devices show a significant reduction in RLS incidence over time.

The incidence of RLS following PFO occluder implantation was accurately measured using TEE at the 1-month, 6-month, and 12-month follow-ups. At the 1-month follow-up, 17.9% of patients exhibited RLS. This percentage showed a significant reduction over time, with only 10.5% of patients displaying RLS at the 6-month follow-up. By the 12-month follow-up, the incidence of RLS had further decreased to a mere 3.4% (Figure 1). Despite a tendency towards a higher incidence of RLSs in the Cardia PFO-Star group, the differences between the groups were not statistically significant (Table 5). Residual shunts were managed conservatively with continued aspirin therapy and echocardiographic monitoring. None of the residual shunts were hemodynamically significant enough to require re-intervention. The majority of residual shunts were observed at the superior or inferior edge of the occluder.

### 3.4. Postprocedural Events

During the first 12 months following PFO occlusion, various postprocedural events were investigated across the cohort, with a closer examination of the differences between the four devices used. Minor bleeding occurred in three patients (0.4%) and vascular access site complications occurred in five patients (0.7%). These were not significantly different among devices. Late-onset pericardial effusion was an uncommon complication, occurring in two patients (0.3%) only. Notably, these cases were exclusively associated with the Cardia PFO-Star device, showing a significant *p*-value of 0.01. There were no instances of pericardial tamponade across any of the devices. Device embolism was a rare event, noted in only one patient (0.1%) who had been implanted with the Cardia PFO-Star device. Similarly, erosions were reported in two patients (0.3%) and were solely linked to the Cardia PFO-Star device (*p* = 0.01).

New onset of atrial fibrillation was the most common complication, affecting 43 patients (6%) overall. The incidence varied among the devices: Occlutech PFO occluder had a rate of 6.6%, Amplatzer PFO occluder 3.1%, Gore septal occluder 8.4%, and Cardia PFO-Star 4.6%, with a *p*-value of 0.07, suggesting a trend towards a higher rate with the Gore septal occluder but not reaching statistical significance.

The incidence of TIA was low and was reported in three patients (0.4%). This event was noted in 0.9% of patients with the Amplatzer PFO occluder and 0.3% with the Gore septal occluder, with no cases in those with Occlutech PFO occluder or Cardia PFO-Star devices (*p* = 0.8). Stroke was another event with an incidence of four cases (0.6%). Similar to TIA, the incidence of stroke did not significantly differ between the four occluder devices.

The incidence of device thrombus was 2.5% and was significantly higher in patients with the Gore septal occluder (5.4%) compared to 1.1% for Cardia PFO-Star, 0.4% for Amplatzer PFO occluder, and none for Occlutech PFO occluder (*p* < 0.001).

Pulmonary embolism was reported in three patients (0.4%) and was exclusively associated with the Amplatzer PFO occluder (*p* = 0.1). Similarly, peripheral thrombi were noted in three patients (0.4%), again all in the Amplatzer group (*p* = 0.1). Importantly, there were no deaths reported in any group (Table 6).

## 4. Discussion

In this study, we aimed to investigate four PFO closure devices (Occlutech PFO occluder, Amplatzer PFO occluder, Gore septal occluder, and Cardia PFO-Star) used in our cohort of 716 patients over a period of 23 years, and evaluate their safety, efficacy and clinical outcomes over a 12-month follow-up period.

The study population was representative of the typical PFO closure patient cohort, with a predominance of younger individuals, consistent with the known epidemiology of PFO [8]. The initial procedural success rate was impressive across all devices, with an overall success rate of 98.9%. This aligns with the findings from the UK registry study, which reported a 99.4% success rate for PFO closure procedures [9]. Our study identified a low incidence of periprocedural complications, including vascular access site complications (0.6%), new-onset atrial fibrillation (0.6%), and hemodynamically significant pericardial effusion (0.3%). These rates are consistent with the safety profiles reported in other studies [10]. The similarity in complication rates across different devices further supports their comparable safety during the periprocedural period.

The incidence of RLS was closely monitored, showing a significant reduction from 17.9% at 1 month to 3.4% at 12 months post-implantation. This decline underscores the effectiveness of the devices in achieving long-term closure of the PFO. Although the Cardia PFO-Star exhibited a tendency towards a higher incidence of RLS, this was not statistically significant, indicating that RLS management is comparable across devices. During a follow-up period of 12 months, seven of our patients experienced recurrent stroke or TIA. Importantly, the rate of these recurrent events was not significantly higher in patients with RLS compared to those without. However, studies with longer follow-up periods (up to 11 years), such as the study by Deng et al., have demonstrated a significantly increased risk of recurrent stroke/TIA in patients with moderate to large RLS, with a reported hazard ratio of 3.05 [11]. These findings highlight the critical need for extended monitoring and follow-up to fully comprehend the longer-term risks associated with RLSs.

The incidence of a few postprocedural events during the first 12 months varied among the devices. Notably, the Cardia PFO-Star was associated with specific complications, such as pericardial effusion and erosions, both of which were not observed with other devices (*p* = 0.01). Erosion occurs when the device irritates or damages surrounding tissue. The friction or constant movement of the device against the atrial wall can lead to tissue breakdown, resulting in erosion. Pericardial effusion can develop if the erosion extends into the pericardium or if inflammation is triggered by the device’s presence, leading to fluid accumulation. The higher incidence of these complications after Cardia PFO-Star occludes might be due to its unique design and the way it anchors to the septum. Over the years, the Cardia PFO-Star has been replaced by its successors, such as the Ultrasept series, which are FDA approved and currently among the most widely deployed devices [12].

New-onset atrial fibrillation/atrial flutter has been reported more often after PFO closure compared to medical therapy (OR 4.40, 95% CI 2.24–8.67) [13]. The mechanism underlying this association is not well understood, but it may be related to the foreign body reaction to the device, irritation of the atrial wall, or incomplete sealing of the defect. This underscores the need for careful patient selection and monitoring when opting for device-based PFO closure. In our cohort, the incidence of new-onset atrial fibrillation/atrial flutter was higher after Gore septal occluder implantation (8.4%), though the *p*-value did not reach statistical significance (*p* = 0.07).

The incidence of device thrombus in our study population (2.5%) aligned with other published studies [14,15,16]. Notably, the Gore septal occluder showed a significantly higher incidence of device thrombus (5.4%, *p* < 0.001), suggesting that it may pose a higher risk for this particular event.

In conclusion, our study provides important insights into the clinical outcomes of different PFO closure devices in a large cohort of patients. Our results suggest that PFO closure is a safe and effective treatment option for selected patients with cryptogenic stroke and PFO. The choice of the most suitable device should be based on careful clinical consideration and the experience of the interventional cardiologist. Further studies, utilizing multicentre randomized designs and extended follow-up periods, are needed to validate our findings.

## 5. The Role of Echocardiographic Guidance in PFO Closure: TEE vs. ICE

Transesophageal echocardiography (TEE) is an essential tool for assessing patent foramen ovale (PFO) and guiding device closure procedures. TEE provides high-resolution imaging of the interatrial septum, allowing clinicians to accurately evaluate the PFO anatomy, including the presence of atrial septal aneurysms or a hypermobile septum. These anatomical features play a crucial role in the selection of the most appropriate occluder device, as they can influence the technical success of the procedure. Additionally, TEE offers real-time guidance during device deployment, ensuring optimal positioning and reducing the likelihood of complications such as residual shunts or device embolization. In our cohort, TEE was the primary imaging modality used for procedural guidance, contributing to the high procedural success rate observed.

Intracardiac echocardiography (ICE), though not utilized in our study, offers an alternative imaging modality with several potential advantages [17]. ICE allows for excellent visualization of the septum and adjacent structures and can be performed under conscious sedation. ICE has been shown to reduce procedure times and minimize radiation exposure, particularly in younger patients, and it can be managed by a single operator [18]. However, ICE also has limitations, such as its higher cost and the need for an additional venous puncture, which could introduce the risk of vascular complications [18]. Future studies may explore the comparative benefits of ICE versus TEE, particularly in terms of procedural efficiency, patient comfort, and long-term outcomes.

## 6. Limitations

There are several limitations in this study. First, as a retrospective analysis from a single center, the findings may not be generalizable to all populations. Second, the follow-up was limited to 12 months, which may not fully capture the long-term outcomes, particularly device-related complications that could manifest beyond this period. Additionally, there may have been variations in operator experience and technique over the 23-year study period, which could affect the results. Finally, the choice of device was not randomized but was instead based on operator discretion, which could introduce selection bias.

## Figures and Tables

**Table 1:** Patients’ basic characteristics.

	Occlutech PFO Occluder	Amplatzer PFO Occluder	Gore Septal Occluder	Cardia PFO-Star	All	*p*-Value
	(*n* = 106)	(*n* = 227)	(*n* = 296)	(*n* = 87)	(*n* = 716)	
Age, mean (SD), years	51.8 ± 11.9	50.4 ± 13.3	50.3 ± 12.4	50.3 ± 12.1	50.6 ± 12.6	0.55
Males, *n* (%)	62 (58.5)	142 (62.6)	178 (60.1)	53 (60.9)	435 (60.8)	0.90
BMI, mean (SD), kg/m^2^	26.2 ± 4.1	25.8 ± 3.8	26.7 ± 4.6	26.0 ± 4.1	26.3 ± 4.3	0.48
Atrial fibrillation/flutter, *n* (%)	2 (1.9)	3 (1.3)	6 (2)	2 (2.3)	13 (1.8)	0.87
Arterial hypertension, *n* (%)	46 (43.4)	96 (42.3)	130 (43.9)	22 (25.3)	294 (41.1)	0.02
Diabetes mellitus, *n* (%)	9 (8.5)	15 (6.6)	19 (6.4)	7 (8)	50 (7)	0.87
Dyslipidemia, *n* (%)	47 (44.3)	85 (37.4)	103 (34.8)	24 (27.6)	259 (36.2)	0.10
History of syncope, *n* (%)	3 (2.8)	5 (2.2)	9 (3.0)	5 (5.7)	22 (3.1)	0.43
Smoking, *n* (%)	34 (32.1)	73 (32.6)	113 (38.2)	25 (28.7)	245 (34.3)	0.30
COPD, *n* (%)	2 (1.9)	4 (1.8)	3 (1)	1 (1.1)	10 (1.4)	0.85
Chronic kidney disease, *n* (%)	1 (0.9)	2 (0.9)	1 (0.3)	1 (1.1)	5 (0.7)	0.51
Serum creatinine, mean (SD), mg/dL	0.82 ± 0.2	0.88 ± 0.5	0.83 ± 0.2	0.84 ± 0.2	0.85 ± 0.3	0.10
hsTnT, median (IQR), pg/mL	6.1 (3.4–8.4)	5.8 (3.0–12.0)	5.0 (4.0–8.5)		6.0 (4.0–9.3)	NA
NT-proBNP, median (IQR), ng/L	101.5 (33.5–247.2)	58.0 (38–126.5)	60.0 (33.0–95.0)		59.5 (33.0–116.0)	NA

BMI = Body Mass Index. COPD = chronic obstructive pulmonary disease. hsTnT = high-sensitive Troponin T. IQR = interquartile range. NA = not applicable. NT-proBNP = N-terminal pro B-type natriuretic peptide.

**Table 2:** Indications for PFO occlusion.

	Occlutech PFO Occluder	Amplatzer PFO Occluder	Gore Septal Occluder	Cardia PFO-Star	All
	(*n* = 106)	(*n* = 227)	(*n* = 296)	(*n* = 87)	(*n* = 716)
Stroke, *n* (%)	77 (72.6)	143 (63)	204 (68.9)	54 (62.1)	478 (66.8)
TIA, *n* (%)	12 (11.3)	48 (21.1)	44 (14.9)	25 (28.7)	129 (18)
Peripheral embolic event, *n* (%)	9 (8.5)	19 (8.4)	11 (3.7)	4 (4.1)	43 (6)
Decompression illness, *n* (%)	0 (0)	1 (0.4)	1 (0.3)	0 (0)	2 (0.3)
Primary prevention, *n* (%)	2 (1.9)	3 (1.3)	12 (4.1)	0 (0)	17 (2.4)
Others, *n* (%)	2 (1.9)	5 (2.2)	6 (2)	2 (2.3)	15 (2.1)

TIA = transient ischemic attack.

**Table 3:** Procedural data.

	Occlutech PFO Occluder	Amplatzer PFO Occluder	Gore Septal Occluder	Cardia PFO-Star	All	*p*-Value
	(*n* = 106)	(*n* = 227)	(*n* = 296)	(*n* = 87)	(*n* = 716)	
Successful implantation, *n* (%)	106 (100)	222 (97.8)	294 (99.3)	86 (98.9)	708 (98.9)	0.3
Procedure duration, mean (SD), min	38.0 ± 18.1	33.7 ± 16.7	37.4 ± 18.7	33.5 ± 10.5	36.2 ± 17.8	0.1
Duration of fluoroscopy, mean (SD), min	5.9 ± 5.3	4.2 ± 5.2	5.6 ± 5.9	3.6 ± 3.0	5.1 ± 5.5	<0.01

**Table 4:** Periprocedural complications.

	Occlutech PFO Occluder	Amplatzer PFO Occluder	Gore Septal Occluder	Cardia PFO-Star	All	*p*-Value
	(*n* = 106)	(*n* = 227)	(*n* = 296)	(*n* = 87)	(*n* = 716)	
Hemodynamically significant pericardial effusion, *n* (%)	0	1 (0.4)	0	1 (1.1)	2 (0.3)	0.15
Device embolism, *n* (%)	0	1 (0.4)	0	0	1 (0.1)	1
New onset of atrial fibrillation, *n* (%)	1 (0.9)	1 (0.4)	1 (0.3)	1 (1.1)	4 (0.6)	0.9
TIA, *n* (%)	0	0	0	1 (1.1)	1 (0.1)	0.3
Stroke, *n* (%)	0	0	0	0	0	NA
Air embolism, *n* (%)	0	1 (0.4)	0	0	1 (0.1)	1
Puncture site complications, *n* (%)	0	1 (0.4)	0	0	1 (0.1)	1
WHO classification of bleeding						
Minor (grades 0–2), *n* (%)	0	2 (0.9)	0	0	2 (0.3)	0.6
Major (grades 3 and 4), *n* (%)	0	1 (0.4)	0	0	1 (0.1)	1
Death, *n* (%)	0	0	0	0	0	NA

TIA = Transient ischemic attack. NA = not applicable.

**Table 5:** Incidence of residual shunt following PFO occluder implantation during the first 12 months.

	Occlutech PFO Occluder	Amplatzer PFO Occluder	Gore Septal Occluder	Cardia PFO-Star	All	*p*-Value
Residual shunt at 1-month follow-up, *n* (%)	21 (20.2)	45 (22.4)	49 (18.4)	13 (31.7)	128 (17.9)	0.2
Residual shunt at 6-month follow-up, *n* (%)	5 (8.1)	10 (10.3)	12 (9.6)	6 (20.7)	33 (10.5)	0.3
Residual shunt at 12-month follow-up, *n* (%)	1 (1.8)	2 (2.5)	5 (4.7)	1 (4.5)	9 (3.4)	0.7

**Table 6:** Postprocedural events during the first 12 months.

	Occlutech PFO Occluder	Amplatzer PFO Occluder	Gore Septal Occluder	Cardia PFO-Star	All	*p*-Value
WHO classification of bleeding						
Minor (grades 0–2), *n* (%)	0	0	3 (1.0)	0	3 (0.4)	0.4
Major (grades 3 and 4), *n* (%)	0	0	0	0	0	NA
Vascular access site complications, *n* (%)	2 (1.9)	1 (0.4)	1 (0.3)	1 (1.1)	5 (0.7)	0.3
Pericardial effusion, *n* (%)	0	0	0	2 (2.3)	2 (0.3)	0.01
Pericardial tamponade, *n* (%)	0	0	0	0	0	NA
Device embolism, *n* (%)	0	0	0	1 (1.1)	1 (0.1)	0.1
Erosions, *n* (%)	0	0	0	2 (2.3)	2 (0.3)	0.01
New onset of atrial fibrillation, *n* (%)	7 (6.6)	7 (3.1)	25 (8.4)	4 (4.6)	43 (6.0)	0.07
New onset of atrial flutter, *n* (%)	1 (0.9)	0	1 (0.3)	0	2 (0.3)	0.6
TIA, *n* (%)	0	2 (0.9)	1 (0.3)	0	3 (0.4)	0.8
Stroke, *n* (%)	1 (0.9)	1 (0.4)	2 (0.7)	0	4 (0.6)	0.9
Device thrombus, *n* (%)	0	1 (0.4)	16 (5.4)	1 (1.1)	18 (2.5)	<0.001
Pulmonary embolism, *n* (%)	0	3 (1.3)	0	0	3 (0.4)	0.1
Peripheral thrombi, *n* (%)	0	3 (1.3)	0	0	3 (0.4)	0.1
Death, *n* (%)	0	0	0	0	0	NA

TIA = Transient ischemic attack. NA = not applicable.

## Data Availability

The original contributions presented in the study are included in the article.
